# Inferring Visuomotor Priors for Sensorimotor Learning

**DOI:** 10.1371/journal.pcbi.1001112

**Published:** 2011-03-31

**Authors:** Edward J. A. Turnham, Daniel A. Braun, Daniel M. Wolpert

**Affiliations:** Computational and Biological Learning Laboratory, Department of Engineering, University of Cambridge, Cambridge, United Kingdom; Northwestern University, United States of America

## Abstract

Sensorimotor learning has been shown to depend on both prior expectations and sensory evidence in a way that is consistent with Bayesian integration. Thus, prior beliefs play a key role during the learning process, especially when only ambiguous sensory information is available. Here we develop a novel technique to estimate the covariance structure of the prior over visuomotor transformations – the mapping between actual and visual location of the hand – during a learning task. Subjects performed reaching movements under multiple visuomotor transformations in which they received visual feedback of their hand position only at the end of the movement. After experiencing a particular transformation for one reach, subjects have insufficient information to determine the exact transformation, and so their second reach reflects a combination of their prior over visuomotor transformations and the sensory evidence from the first reach. We developed a Bayesian observer model in order to infer the covariance structure of the subjects' prior, which was found to give high probability to parameter settings consistent with visuomotor rotations. Therefore, although the set of visuomotor transformations experienced had little structure, the subjects had a strong tendency to interpret ambiguous sensory evidence as arising from rotation-like transformations. We then exposed the same subjects to a highly-structured set of visuomotor transformations, designed to be very different from the set of visuomotor rotations. During this exposure the prior was found to have changed significantly to have a covariance structure that no longer favored rotation-like transformations. In summary, we have developed a technique which can estimate the full covariance structure of a prior in a sensorimotor task and have shown that the prior over visuomotor transformations favor a rotation-like structure. Moreover, through experience of a novel task structure, participants can appropriately alter the covariance structure of their prior.

## Author Summary

When learning a new skill, such as riding a bicycle, we can adjust the commands we send to our muscles based on two sources of information. First, we can use sensory inputs to inform us how the bike is behaving. Second, we can use prior knowledge about the properties of bikes and how they behave in general. This prior knowledge is represented as a probability distribution over the properties of bikes. These two sources of information can then be combined by a process known as Bayes rule to identify optimally the properties of a particular bike. Here, we develop a novel technique to identify the probability distribution of a prior in a visuomotor learning task in which the visual location of the hand is transformed from the actual hand location, similar to when using a computer mouse. We show that subjects have a prior that tends to interpret ambiguous information about the task as arising from a visuomotor rotation but that experience of a particular set of visuomotor transformations can alter the prior.

## Introduction

Uncertainty poses a fundamental problem for perception, action and decision-making. Despite our sensory inputs providing only a partial and noisy view of the world, and our motor outputs being corrupted by significant amounts of noise, we are able to both perceive and act on the world in what appears to be an efficient manner [1], [2]. The investigation of the computational principles that might underlie this capability has long been of interest to neuroscientists, behavioral economists and experimental psychologists. Helmholtz [3] was one of the first to propose that the brain might operate as an ‘inference machine’ by extracting perceptual information from uncertain sensory data through probabilistic estimation. This computational framework has now gained considerable experimental support and has recently led to the formulation of the ‘Bayesian brain’ hypothesis [4], [5]. According to this hypothesis, the nervous system employs probabilistic internal models representing Bayesian probabilities about different states of the world that are updated in accordance with Bayesian statistics whenever new evidence is incorporated. Crucially, this update depends on two components: a *prior* that represents a statistical distribution over different possible states of the world, and the incoming *evidence* about the current state that is provided through noisy sensory data.

In the Bayesian framework the prior can have a strong impact on the update, with particular priors leading to inductive biases when confronted with insufficient information. Many perceptual biases have been explained as the influence of priors learned from the statistics of the real world, such as the prior for lower speed when interpreting visual motion [6], [7], the prior for lights to shine from above when interpreting object shape [8], [9] and the prior that near-vertical visual stimuli are longer than horizontal stimuli [10]. However, there are some phenomena such as the size-weight illusion – the smaller of two objects of equal weight feels heavier – that appear to act in the direction opposite to that expected from straightforward integration of the prior with sensory evidence [11], [12]. Interestingly, despite the perceptual system thinking the smaller object is heavier, the motor system is not fooled as, after experience with the two objects, people generate identical forces when lifting them [13]. Many cognitive biases can also be explained, not as errors in reasoning, but as the appropriate application of prior information [14]–[16], and the Bayesian approach has been particularly successful in explaining human performance in cognitive tasks [17], [18].

In sensorimotor tasks, a number of studies have shown that when a participant is exposed to a task which has a fixed statistical distribution they incorporate this into their prior and combine it with new evidence in a way that is consistent with Bayesian estimation [5], [19], [20]. Similarly, when several sources of evidence with different degrees of uncertainty have to be combined, for example a visual and a haptic cue, humans integrate the two sources of evidence by giving preference to the more reliable cue in quantitative agreement with Bayesian statistics [21]–[23]. Moreover, computational models of motor control, such as optimal feedback control [24]–[27], are based on both Bayesian estimation and utility theory and have accounted for numerous phenomena in movement neuroscience such as variability patterns [24], bimanual movement control [28], [29], task adaptation [30]–[32] and object manipulation [33]. There have also been several proposals for how such Bayesian processing may be implemented in neural circuits [34]–[36].

If one uses Bayesian estimation in an attempt to learn the parameters of a new motor task, the prior over the parameters will impact on the estimates. While previously priors have been either imposed on a motor task or assumed, there has been no paradigm that allows the natural prior distribution to be assessed in sensorimotor tasks. Here we develop a technique capable of estimating the prior over tasks.

We examine visuomotor transformations, in which a discrepancy is introduced between the hand's actual and visual locations, and estimate the prior over visuomotor transformations. Importantly, we are not simply trying to estimate the mean of the prior but its full covariance structure. Subjects made reaching movements which alternated between batches in which feedback of the hand's position was either veridical or had a visuomotor transformation applied to it. By exposing participants to a large range of visuomotor transformations we are able to fit a Bayesian observer model to estimate the prior. Our model assumes that at the start of each transformation batch a prior is used to instantiate the belief over visuomotor transformations and this is used to update the posterior after each trial of a transformation batch. The prior to which the belief is reset at the start of a transformation trial may change with experience. For our model we estimate the average prior used over an experimental session by assuming it is fixed within a session, as we expect the prior to only change slowly in response to the statistics of experience.

Our approach allows us to study the inductive biases of visuomotor learning in a quantitative manner within a Bayesian framework and to estimate the prior distribution over transformations. Having estimated the prior in one experimental session, we examine whether extensive training in two further sessions with a particular distribution of visuomotor transformations could alter the participants' prior.

## Results

**Figure 1:**
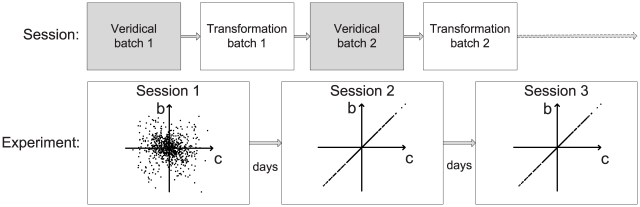
The experimental design. Each session alternated between veridical and transformed batches of trials. Each subject participated in three sessions, the first using an uncorrelated distribution of transformations, and the second and third using a correlated distribution. The joint distributions of 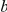 and 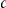 are plotted.

Subjects made reaching movements to targets presented in the horizontal plane, with feedback of the hand position projected into the plane of movement by a virtual-reality projection system only at the end of each reach (terminal feedback). Reaches were from a starting circle, 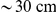 in front of the subject's chest, to a target randomly chosen from within a rectangle centred 11 cm from the starting circle (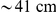 in front of the chest). Subjects made reaching movements in batches which were alternately veridical and transformed (Figure 1 top, see Methods for full details). In a veridical batch, the cursor was always aligned with the hand. In a transformation batch, subjects experienced a visuomotor transformation that remained constant throughout the batch and in which the terminal-feedback cursor position (**v**) was a linear transformation (specified by transformation matrix **T**) of the final hand position (**h**) relative to the (constant) starting point of the reaches: 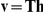. In component form, this can be written as
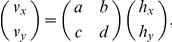



where we define the 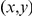 coordinates as (left-right, backward-forwards) relative to the subject. Each transformed batch used a different transformation. The number of transformations experienced was at least 108 for each subject in each of three experimental sessions (mean 147 transforms, 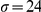; see Table 1). Transformation batches contained at least three trials (mean length: 4.9 trials, 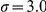) and generally continued until a target had been hit (achieved on 91% of batches). Veridical batches always continued until a target had been hit (mean length: 1.4 trials, 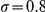). The purpose of the veridical batches was to wash out short-term learning. Transformed trials were distinguished from veridical trials by the color of the targets, so that the onset of a new transformation was clear to the subjects. The length of a session was on average 921 trials (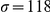) and lasted 82 minutes (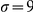). Subjects performed three experimental sessions on different days. The transformations used in Session 1 were drawn from an ‘uncorrelated’ distribution so as to minimize pairwise correlations between elements of the transformation matrix. The transformations used in Session 2 & 3 were drawn from a ‘correlated’ distribution to examine whether this would change subjects' priors (see Figure 1 bottom).

**Table 1:** The experimental subjects.

	Session 1			Session 2			Session 3	
Subject	Transforms	Trials	Delay	Transforms	Trials	Delay	Transforms	Trials
1	120	745	3	118	786	9	120	850
2	150	947	3	150	830	8	200	1102
3	144	827	4	150	860	8	180	977
4	133	944	3	140	929	9	160	1075
5	150	871	5	150	838	8	206	1076
6	140	970	6	124	928	9	155	1117
7	160	1090	5	151	1035	7	144	955
8	133	861	3	108	731	7	134	762

The number of transformations and trials in each experimental session, and the lengths of the delay in days between sessions.

### Initial analysis

**Figure 2:**
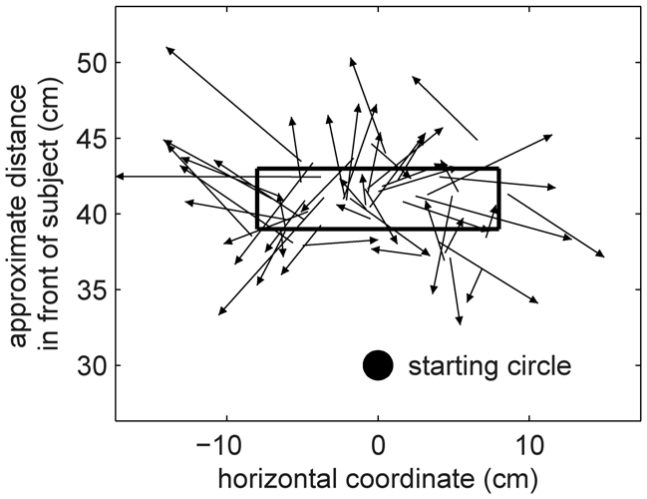
Target area and example perturbation vectors. The starting point of the reaches (1 cm radius circle) and the area from which the centres of targets were drawn (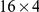 cm rectangle: not displayed to the subject) are shown, in addition to ‘perturbation vectors’ from subjects' hand positions to the corresponding cursor positions on the first trials of 50 example transformations from Session 1.

**Figure 3:**
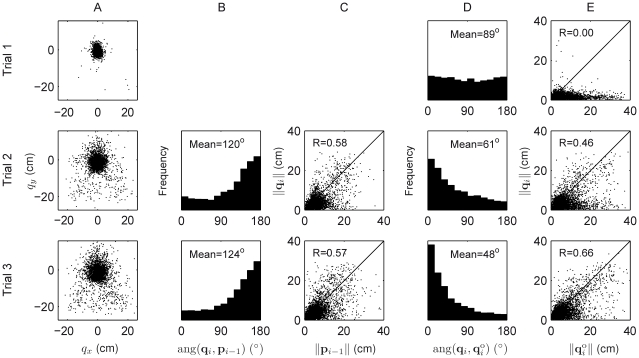
Analysis of hand positions across the trials of a transformation batch. Column **A** shows the distribution (across all subjects and sessions) of the ‘target-hand vector’ representing the position of the hand relative to the target, 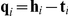, separately for trials 1, 2 & 3 of a transformation batch. Columns **B** and **C** show the relation between the target-hand vector and the ‘perturbation vector’ from hand to cursor on the previous trial, 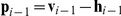. Column B gives the distribution of the angle between the two vectors, and Column C plots the lengths of the vectors against each other. Columns **D** and **E** make the same comparisons between the target-hand vector and the target-hand vector that would place the cursor on the target, 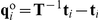. Column D gives the distribution of the angle between the two vectors, and Column E plots the lengths of the vectors against each other.

Figure 2 shows the starting location and rectangle in which the targets could appear together with 50 examples of ‘perturbation vectors’ that join the hand position on the first trial of a transformation batch to the displayed cursor position (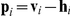 where 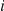 is the trial index, in this case 1). On the first trial of each transformation batch, the ‘target-hand vector’ joining the centre of the target 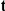 to the final position of the hand 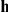 (the ‘target-hand vector’ 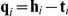) was shorter than 3 cm in 90% of cases (Figure 3, column A, top panel), suggesting that the preceding veridical batches had washed out most of the learning. Subjects were instructed that on the second and subsequent trials of each transformation batch, they should attempt to compensate for the transformation in order to hit the target with the cursor. Hence on trials 2 and 3, the proportion of final hand positions within 3 cm of the target drops to 43% (middle panel of Figure 3, column A) and 36% (bottom panel), respectively. Further analysis suggests that the increase in length of the target-hand vectors on trials 2 and 3 is due to subjects attempting to counter the transformation, rather than just exploring the workspace randomly. Figure 3, column B shows that the direction of the target-hand vector tends to be opposite to that of the perturbation vector experienced on the previous trial, while column C shows that the lengths of these two vectors are positively correlated. The ratio of the length of the target-hand vector on the second trial to that of the perturbation vector on the first trial gives a measure of the extent of the adaptation induced by the experience on the first trial, with a value of zero suggesting no adaptation. We regressed this adaptation measure for all subjects and sessions (removing a few outliers – 0.34% – where this measure was greater than 5) against the absolute angular difference between the direction of the first and second targets, in order to test the assumption made later in our modelling that adaptation generalizes across the workspace. If there were a local generalization function with a decay based on target direction we would expect that the greater the angular difference the smaller the adaptation measure. The fit had a slope which was not significantly different from zero (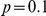) suggesting global generalization.

Compensatory responses tend to be in the correct direction: Column D shows that target-hand vectors on trials 2 and 3 tend to be in the same direction as the target-hand vector that would place the cursor on the target (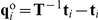), and column E shows that the lengths of these two vectors are also positively correlated. This suggests that subjects are adapting within a batch so as to compensate for the induced perturbation.

### Bayesian observer model

**Figure 4:**
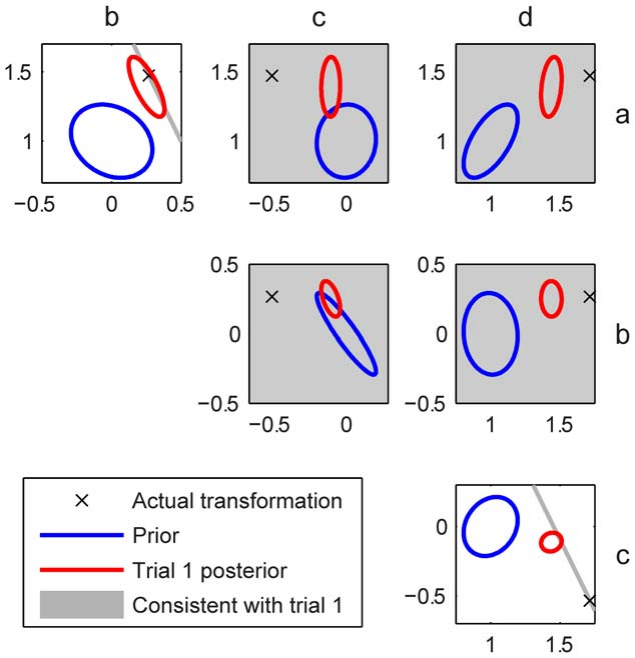
Schematic of the Bayesian observer model. The plots show six 2-dimensional views of the 4-dimensional probability space of the 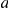, 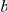, 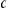 & 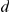 parameters of the transformation matrix. The Gaussian prior is shown in blue (marginalised 1 s.d. isoprobability ellipses). On the first trial the evidence the subject receives (for simplicity shown here as noiseless) does not fully specify the transformation uniquely, and the transformations consistent with this evidence are shown in gray. This evidence (as a likelihood) is combined with the prior to give the posterior after the first trial (red ellipses: these are shown calculated from the noisy visual feedback) and the MAP of this posterior is taken as the estimate of the transformation. The cross shows the position of the actual transformation matrix used in generating the first-trial evidence.

We fit subjects' performance on the first two trials of each transformed batch using a Bayesian observer model in which we assume subjects attempt to estimate the four parameters (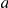, 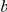, 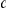, & 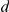) of the transformation matrix. We represent the subject's prior as a four-dimensional multivariate Gaussian distribution over these four parameters, centred on the identity transformation (since subjects naturally expect the visual location of the hand to match its actual location). Our inference problem is to determine the 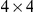 covariance matrix of this prior. Figure 4 includes a schematic of a prior with the four-dimensional distribution shown as six two-dimensional marginalizations with isoprobability ellipses (blue), representing the relation between all possible pairings of the four elements of the transformation matrix.

An optimal observer would integrate this prior with information received on the first trial (hand position and visual feedback of hand position) to generate a posterior over transformations. Even if there were no noise in proprioception or vision, the information from the first trial would not uniquely specify the underlying transformation. For example, for a particular feedback on the first trial the evidence is compatible with many settings of the four parameters (grey lines and planes in Figure 4). Therefore, given the inherent ambiguity (and noise in sensory inputs), the estimated transformation depends both on the sensory evidence and prior which together can be used to generate a posterior distribution over the four parameters of the transformation matrix (Figure 4, red ellipses). Our Bayesian observer then uses the most probable transformation (the MAP estimate is the centre of the red ellipses in Figure 4) to determine where to point on the second trial. Our aim is to infer the prior distribution for each subject in each experimental session by fitting the pointing location on the second trial based on the experience on the first trial. The model assumes the observer starts each transformation batch within a session with the same prior distribution, although this distribution will of course be updated during each batch by combination with evidence. As shown above, these updates are washed out between batches through the interleaved veridical batches.

### Session 1

**Figure 5:**
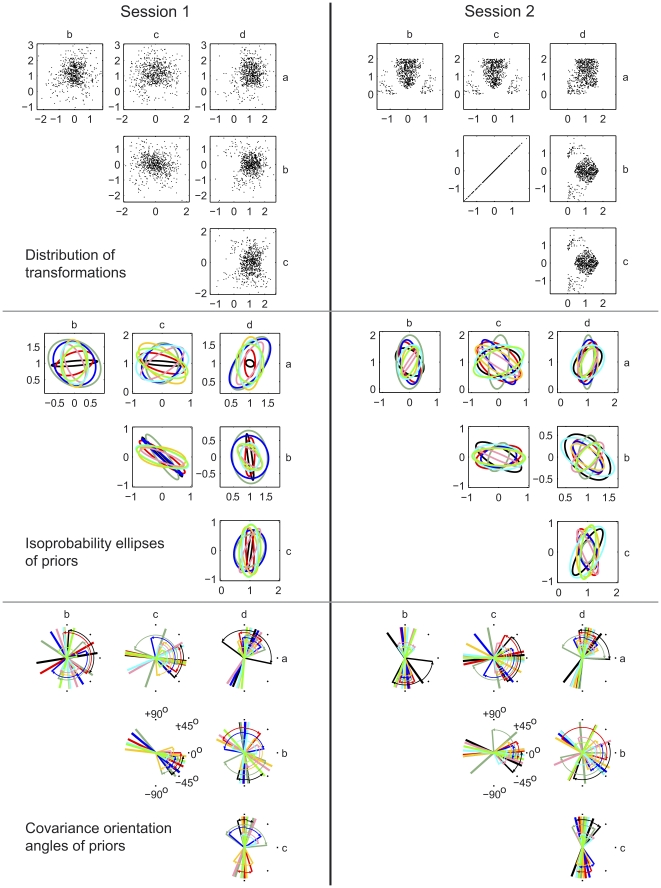
Distributions of transformations and prior distributions in Sessions 1 and 2. Left column: Session 1. Right column: Session 2. Top row: the distributions of transformations in the two sessions. In each case 700 of the experimental transformations are plotted in the six projections of the 4-D space of linear transformations used in Figure 4. Middle row: the priors fit to the data of the 8 subjects, plotted in the style used for the priors in Figure 4. Each covariance matrix has been scaled so that its largest eigenvalue is unity, in order that all priors can be displayed together without any being too small to see. Bottom row: confidence limits on covariance orientation angles, shown for each pairing of the four elements of the transformation matrix 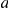, 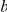, 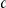, 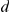. These confidence limits were obtained by bootstrapping, as explained in Methods. For each subject, thick lines show the mean angle across the 1000 or more resampled fits. Thin lines, connected to the mean line by curved arrows, give the 95% confidence limits. Only the range 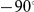 to 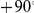 is labelled, because the data is axial and therefore only exists in a 180 range.

In Session 1, transformations were sampled so as to minimize pairwise correlations between elements of the transformation matrix. This ‘uncorrelated’ distribution was designed to avoid inducing learning of new correlations. The set of transformations experienced in the first session is shown in the top-left cell of Figure 5, viewed in the same six projections of the four-dimensional space used in Figure 4. The Gaussian priors fit to each of the eight subjects' data in Session 1 are shown in the middle-left cell of Figure 5. For some pairs of elements of the transformation matrix (e.g. 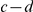) the prior appears to show little correlation whereas for others (e.g. 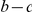) there appears to be a stronger correlation. To quantify these relations we examined the correlation coefficients between each pair of elements of the transformation matrix across the subjects. First, to examine the consistency of the correlation across subjects we tested the null hypothesis that subjects' correlation coefficients were uniformly distributed between 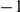 and 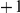 (Kolmogorov-Smirnov test). We found that only between elements 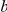 and 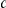 was the correlation significantly consistent (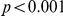). In addition we used a t-test to examine whether the correlations across subjects were significantly different from zero (although correlations are strictly speaking not normally distributed). We found that only the 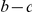 correlation was significant (mean 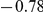, 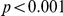).

We also analyzed the orientations of these covariance ellipses. Confidence limits on the orientation angle of the long axis of each ellipse were obtained by bootstrapping. The bottom-left cell of Figure 5 shows, for each subject, the mean angle (thick line) and the 95% confidence limits (thin lines connected by curved arrows). The 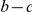 confidence limits are exclusively in the negative range for all but two subjects, while for all other pairings of elements confidence limits for most subjects overlap the 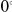 or 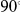 points indicative of an absence of correlation. The mean 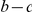 angle *across* subjects was 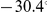 (95% confidence limits obtained by bootstrapping of the best fits: 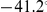 to 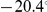). We also found that the 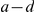 covariance angle was significantly positive (mean across subjects 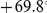, confidence limits 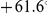 to 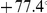).

### Sessions 2 and 3

Each subject participated in Session 2 between three and six days after Session 1, and in Session 3 between seven and nine days after Session 2 (Table 1). These sessions both used a set of transformations whose distribution was chosen so as to be very different from the subjects' priors measured in Session 1. This allowed us to examine whether we could change subjects' priors through experience. As subjects had priors with a strong negative correlation between elements 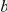 and 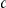 of the transformation matrix we used a ‘correlated distribution’ over transformations in which the 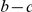 correlation was set to 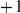, with an orientation angle of 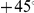 (Figure 5, top-right cell). Importantly, the two distributions used in Session 1 and in Sessions 2 & 3 were designed so that the distribution of evidence (that is the relation between visual and actual hand locations) shown on the first trial of each transformation batch was identical under the two distributions (see Methods). Therefore any changes in behavior on the second trial (which we use to estimate the prior) arose because of changes in the subject's prior. The remainder of the trials within a batch have different statistics between Session 1 and Sessions 2 & 3, so we did not use data beyond trial 2 to estimate the prior, although this could be used by the subjects to alter their internal prior.

The priors fit to the data of the five subjects in Session 2 are shown in the middle-right cell of Figure 5. We found that in Session 2 the 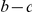 correlations across subjects were now not significantly different from zero (mean correlation coefficient 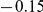, 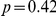, t-test) and were not distributed significantly non-uniformly across subjects (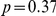, K-S test). Confidence limits (Figure 5, bottom-right cell) on the 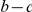 covariance angle now overlapped 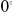 for all but one subject, again implying the absence of correlation. Confidence limits on the mean 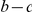 covariance angle across subjects overlapped 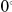 (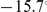 to 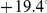, mean 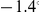). A weak but significant 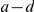 correlation was now found (mean 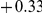, 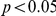 on t-test and K-S test), and the 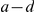 covariance angle continued to be positive (mean 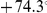, confidence limits 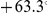 to 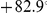), although angles were not significant for any individual subject.

**Figure 6:**
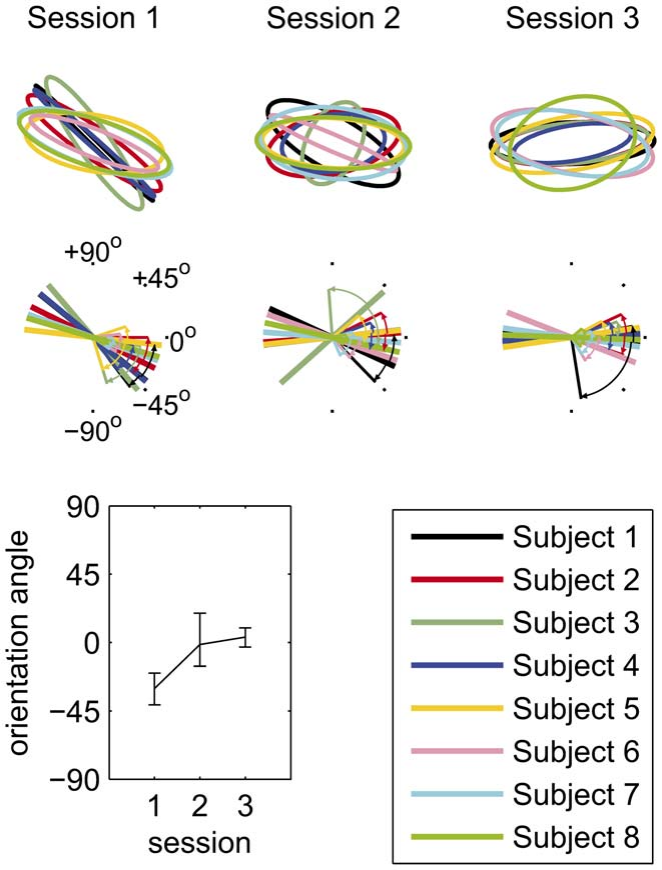
Evolution of the *b*-*c* relationship. The top line shows the best fits in each of the experimental sessions, for each of the eight subjects; the middle line shows means and confidence limits on the covariance orientation angles. The bottom-left graph shows the mean across subjects of the orientation angles from the best fits to each subject's data, with 95% confidence limits on the mean found by bootstrapping.

In Session 3 (see Figure 6, which summarises changes in the 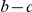 relation across sessions) the 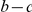 correlation was still not significant (mean correlation coefficient 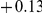, 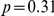 on t-test and 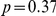 on K-S test). The covariance angle confidence limits now overlapped zero within all subjects and across subjects (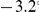 to 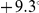, mean 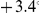). A weak but significant 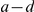 correlation was again found (mean 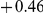, 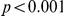 on t-test and 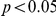 on K-S test), and the 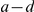 covariance angle continued to be positive (mean across subjects 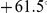, confidence limits 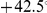 to 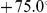), although angles were only significant for three individual subjects.

### Model comparison

To assess the extent to which our Bayesian observer model explained the data, we compared the magnitudes of its errors in predicting hand positions to the errors made by four other models: (A) the ‘no-adaptation’ model, which assumes the hand hits the centre of the target on all trials; (B) the ‘shift’ model, which is also a Bayesian observer but assumes the transformation is a translation; (C) the ‘rotation & uniform scaling’ model, another Bayesian observer that assumes the transformation is a rotation combined with a scaling; (D) the ‘affine’ model, which is a Bayesian observer more general than the standard model in that it accounts for linear transformations combined with shifts. Comparisons of hand position prediction error were made for each trial of a transformed batch from the 2nd to the 7th, although it should be remembered that trials after the 3rd represent progressively fewer batches, with only 44% of batches lasting to the 4th trial and only 19% lasting to the 7th. The Bayesian observer models integrated information about a transformation from all previous trials of a batch when making a prediction for the next trial. Since the Bayesian observer models were all fit to data from the second trials of each transformed batch (i.e. the standard model used the fits presented above), comparison of prediction errors on the second trials themselves was done using 10-fold cross-validation for these models, in order to avoid over-fitting by complex models.

**Figure 7:**
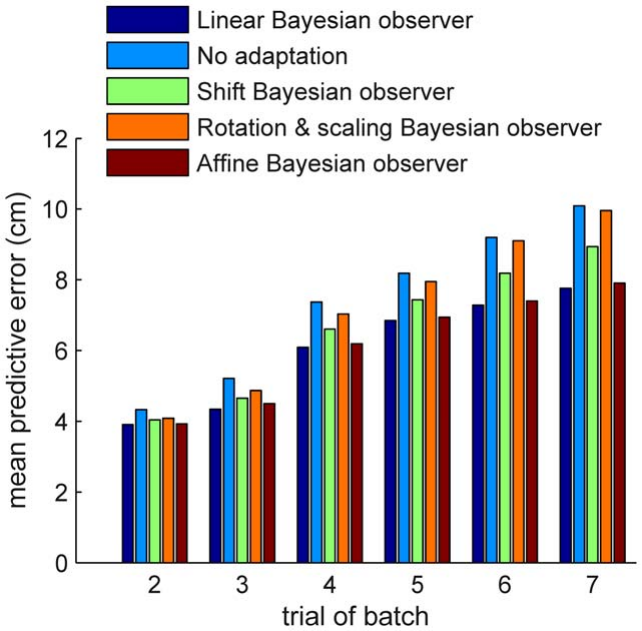
Comparison of standard linear model against other plausible models. Models are compared on the basis of their mean error, across subjects and sessions, in predicting subjects' hand positions on trials 2–7 of transformation batches. For each trial, all batches that lasted for at least that number of trials are used. Errors are capped at 20 cm before averaging, to reduce the effect of outliers. Trial 2 values are computed using 10-fold cross-validation, and later trial values are computed using fits to all transformation batches.

To compare the models we focus on trial 3, which is late enough that the subjects have received a considerable amount of information about the transformation (just enough to specify the whole transformation matrix, in noiseless conditions) but early enough that all batches can be included. Figure 7 shows that on this trial the standard model makes smaller prediction errors for the hand positions (averaged across all sessions) than any other model. The next-best is the affine model (mean error 4.50 cm, versus 4.34 for the linear model). On all other trials, the linear model is also superior to all other models. The failure of the affine model to perform better than the standard model shows that its extra complexity, which allows it to account for shifts, is not necessary. Accounting for shifts made little difference to the linear components of the fits: the correlation coefficients between pairs of elements of the transformation matrix were very similar to those in the linear model fits (median absolute difference across all pairs: 0.11), and the 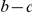 coefficients were again significantly negative in Session 1 (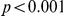 on t-test and Kolmogorov-Smirnov test) and ceased to be significantly different from zero in Sessions 2 and 3. The covariance angles between pairs of elements were also very similar to those in the linear model fits (median absolute difference: 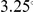), and the 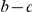 angles were significantly negative in Session 1 (95% confidence limits: 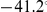 and 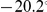) and ceased to be significantly negative in Sessions 2 and 3.

**Figure 8:**
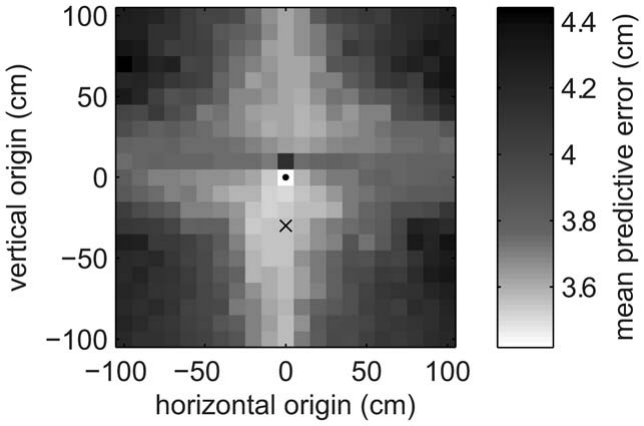
Comparison of possible linear transformation origins for the Bayesian observer model. For each small square the shading denotes the performance of the standard Bayesian observer model when the origin of the linear transformations is set to the centre of that square. Performance is measured using the error between modelled and measured second-trial hand positions, averaged within an experimental session for one subject (after capping all errors at 20 cm) and then averaged across all subjects and all sessions. The small circle shows the start point of the reaches, which is used as the origin in all other modelling. The cross shows the approximate position of the eyes (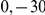 cm).

We also varied the origin of the linear transformations that we used in the Bayesian observer model, to see if the coordinate system used by the experimental subjects was based around the starting point of the reaches (small circle in Figure 8), or about some other location such as the eyes (cross in Figure 8). The shading in Figure 8 represents the fitting error and shows that using the starting point of the reaches as the origin fits the data considerably better than any other position tested (mean error: 3.49 cm for the starting point, versus 3.61 cm for the next best position). In particular, a repeated-measures ANOVA (using subject number and session as the other two factors) shows that using the starting point as origin gives significantly lower errors than using the eye position (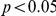).

## Discussion

By exposing participants to numerous linear transformations (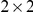 transformation matrices) in a virtual-reality reaching task in the horizontal plane we were able to estimate the prior subjects have over visuomotor transformations. After a new transformation had been experienced for a single trial, we fit the prior in a Bayesian observer model so as to best account for the subsequent reach. That is, for the subject the first reach provides a likelihood which together with his prior leads to a posterior over visuomotor transformations, the maximum of which determines his second reach. While the mean of the prior is assumed to be the identity transformation (vision of the hand is expected to be where the hand really is), we found the estimated prior to have a covariance structure with a strong negative correlation between the off-diagonal elements of the transformation matrix. We then exposed the participants in two further sessions to visuomotor transformations from a distribution that had a positive correlation between these off-diagonal elements (hence the opposite correlation structure to the prior), and remeasured the prior. The estimated prior had changed significantly in that there was now no correlation between the off-diagonal elements, demonstrating learning.

Our study has three key novel features. First, we have developed a technique which can, unlike previous paradigms, estimate the full covariance structure of a prior in a sensorimotor task. Second, we have shown that for our task the prior over visuomotor transformations favors rotation-like structures. Third, we have shown that through experience of a novel correlation structure between the task parameters, participants appropriately alter the covariance structure of their prior.

### Measuring the prior

Previous studies have attempted to determine the natural co-ordinate system used for visuomotor transformations. The dominant paradigm has been to expose subjects to a limited alteration in the visuomotor map and examine generalisation to novel locations in the workspace. These studies show that when a single visual location is remapped to a new proprioceptive location, the visuomotor map shows extensive changes throughout the workspace when examined in one-dimensional [37]–[40] and in three-dimensional tasks [41]. These studies are limited in two ways in their ability to examine the prior over visuomotor transformations. First, they only examine how subjects generalize after experiencing one (or a very limited set of) alterations between visual and proprioceptive inputs. As such the results may depend on the particular perturbation chosen. Second, while the generalization to novel locations can provide information about the co-ordinate system used, it provides no information about the covariance structure of the prior. Our paradigm is able to address both these limitations using many novel visual-proprioceptive mappings to estimate the full covariance structure of the prior over visuomotor transformations.

To study this covariance structure in the fitted priors, we analyzed both the correlation coefficients between elements of the transformation matrix – as a measure of the strength of the relationship between elements – and also the orientation of the covariance ellipses of pairs of elements – as a measure of the slope of the relationship. A significant strong negative correlation was seen between the off-diagonal elements of the 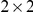 transformation matrices in the priors found in Session 1. Such a relation is found in a rotation matrix, 
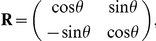



as this corresponds to 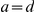 and 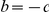 in our transformation matrix. This similarity suggests a bias for subjects to interpret transformations as conforming to rotation-like structures. The 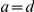 and 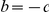 relations would still exist if a rotation were combined with a uniform scaling. We do not claim that subjects believe the transformations to be only rotations and uniform scalings. If they did, we should have found a 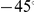 relationship between 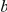 and 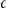 in the prior and a strong 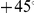 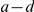 relationship, but the 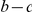 covariance angle was around 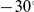 and the 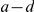 correlation was weak. Rather, it seems likely that the subjects believed many of the transformations in Session 1 to be rotations combined with other perturbations.

Vetter and colleagues [41] also found an apparent bias for rotations. However, these were rotations about the eyes, whereas the centre of the coordinate system in our model is the starting circle, approximately 30 cm in front of the eyes. We showed that our subjects' data across all sessions is best explained using the starting circle as the origin of transformations, rather than the eyes or any other location (Figure 8). The two studies are not contradictory, because our subjects were shown the cursor on top of the start circle at the start and end of every trial, and so would have been likely to learn that it was the origin of the transformations.

Importantly, to measure the prior we ensured that the distribution of transformations in the first session was relatively unstructured in the space of the four elements of the transformation matrix, and in particular the distribution of transformations used had only a very small correlation between the off-diagonal elements. Therefore, it is unlikely (particularly given the adaptation results discussed below) that the prior for rotations came about because of the particular set of transformations used in our paradigm.

Our approach of probing a subject's prior with many transformations would be disrupted if the learning of these transformations interfered with each other. Many studies have shown interference between the learning of similar but opposing visuomotor perturbations [42]–[44], similar to that found between two dynamic perturbations [45], [46]. However, subjects in those experiments were trained for dozens of trials on each perturbation; learning of individual transformations over just a few trials in our experiment would have been much less resilient to overwriting with new memories. Additionally, the veridical batches between each transformation in our experiment would have washed out any *perceptual* or *non-cognitive* component of learning [38], [47]–[50].

The previous work on visuomotor generalization cited above [37]–[39], [41], which found that experiencing single visual-proprioceptive pairs induced remapping throughout the workspace, justifies the assumption made in the analysis of the current study that perturbations experienced at one location will induce adaptive responses throughout the workspace. In addition, our analysis shows that the magnitude of the adaptive response on the second trial does not decrease with the angular deviation of the second target from the first, providing further support for global generalization under terminal feedback. Another reaching study [51] found much more limited generalization across locations, but was criticized [41] on the grounds that the starting point of reaches was not controlled, and that subjects were constrained to make unnatural reaching movements at the height of the shoulder. Work with visual feedback of the hand position throughout the reach has found that scalings are generalized throughout the workspace but rotations are learned only locally [52]. This lack of generalization is clearly at odds with the weight of evidence from terminal-feedback studies. The difference is perhaps due to differing extents of cognitive adaptation under the two feedback conditions.

### Altering the prior

Recent studies have shown that when exposed to tasks that follow a structured distribution, subjects can learn this structure and use it to facilitate learning of novel tasks corresponding to the structure [53]. In the current study, when participants were exposed to a structured distribution of transformations in Sessions 2 & 3 we found that participants' priors changed to become closer to the novel distribution. The estimated prior's negative correlation between the off-diagonal elements observed in the Session 1 priors was abolished by training on a distribution of transformations in which these off-diagonal elements were set to be equal and therefore perfectly positively correlated. This abolition in the fitted priors is evidenced both by the orientations of the covariance ellipses between the off-diagonal elements, which became clustered around 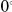, and by the correlation coefficients for this pair of elements, which also clustered around zero. Importantly, the perturbations on the first reach of each transformed batch in Sessions 2 & 3 were generated identically to those in Session 1 so that we can be sure it is the prior that has changed, as the evidence shown to the subject was identically distributed and only varied in terms of the feedback on the second and subsequent trials.

Previous studies have also demonstrated the ability of people to learn priors over novel sensorimotor tasks. For instance, one study showed that subjects learned a non-zero-mean Gaussian prior over horizontal shifts [19], while reaction-time studies [54] succeeded in teaching subjects non-uniform prior distributions over potential targets for a saccade. Similarly, other studies have shown that priors, such as the relation between size and weight [55] and over the direction of light sources in determining shape from shading [8], can be adapted through experience of a training set which differs from the normal prior. In many of these previous studies only the mean of the learned prior was measured, and the priors were generally one-dimensional whereas in the current study we expose subjects to distributions in which there is a novel and multi-dimensional covariance structure. This difference in dimensionality may also explain why a one-dimensional structure of visuomotor rotations [53] could perhaps be learned faster than the three-dimensional structure of transformations used in Sessions 2 & 3 in the present study, which was never learned fully. As dimensionality increases, the amount of data required by a subject to specify the structure increases dramatically.

### Extensions of the technique

In the current study we have made a number of simplifying assumptions which facilitated our analysis but which we believe in future studies could be relaxed. First, we have analysed the prior within the Cartesian coordinate system in which the prior is over the elements of the set of 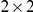 transformation matrices. We believe this coordinate system to be a reasonable starting point for such research, since the visuomotor generalization studies cited above found visuomotor generalization to be linear [37], [38], [41]. In particular, the bias seems to be for rotations [41] rather than shifts in Cartesian space, which are not linear transformations; some studies describe generalization of shifts but as they either only examine a one-dimensional array of targets [37], [38] or a single generalization target [56] their results can not distinguish between rotations and shifts.

Furthermore, the comparison of different models in this paper (Figure 7) shows that our linear-transformations model performs better than a more complex affine-transformations model and simpler models such as the shift model. This suggests that our linear-transformations model is of the right level of complexity for explaining subjects' performance in this paradigm. That the shift model performed considerably better than the no-adaptation model does not show that subjects believed any transformations to have a shift component and that the extra complexity of the affine-transformations model is therefore necessary. Rather, the shift model may have simply managed to approximate linear transformations (such as small rotations) as shifts.

A further simplifying assumption was that the prior takes on a multivariate Gaussian distribution over elements of the transformation matrix. The true prior could be both nonlinear and non-Gaussian in our parameterization and as such our estimation may be an approximation to the true prior. While it may be possible to develop techniques to find a prior which has more complex structure, such as a mixture of Gaussians, such an analysis would require far more data for the extra degrees of freedom incurred by a more complex model.

Another model assumption is that the subject uses the MAP transformation to choose his hand position. Although it is common for Bayesian decision models to use point estimates of parameters when making decisions, different rules that also take into account the observer's uncertainty over the transformation may better model the data.

Our model was purely parametric, with the observer performing inference directly over the parameters of the transformation matrix. In the future it will be interesting to consider hierarchical observer models which would perform inference over *structures* of transformations, such as rotations, uniform scaling or shearings, and simultaneously over the parameters within each structure, such as the angle of the rotation. This observer would have a prior over structures and over the parameters within each structure. Nevertheless, our study shows that we can estimate the full covariance structure of a prior in a sensorimotor task, that this prior has similar form across subjects and that it can be altered by novel experience.

## Methods

### Experimental methods

All eight subjects were naïve to the purpose of the experiments. Experiments were performed using a vBOT planar robotic manipulandum [57]. Subjects used their right hand to grasp the handle, which they could move freely in the horizontal plane. A planar virtual reality projection system was used to overlay images into the plane of movement of the vBOT handle. Subjects were not able to see their arm.

#### Ethics statement

All subjects gave written informed consent in accordance with the requirements of the Psychology Research Ethics Committee of the University of Cambridge.

#### First session

In the first session, subjects alternated between making reaching movements under veridical and transformed feedback (see Figure 1 for a summary of the experimental design). On each trial subjects made a reach from a midline starting circle (1 cm radius, 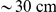 in front of the subject's chest) to a visually presented target. To initiate a trial the hand had to be stationary within the starting circle (speed less than 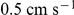 for 800 ms), at which point the visual target (2 cm radius) appeared. The target location was selected pseudorandomly from a 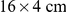 rectangle centred 11 cm further in front of the subject's chest than the starting location (see Figure 2). In the veridical batches, visual feedback of the final hand location (0.5 cm radius cursor) was displayed for 1 s at the end of the movement (hand speed less than 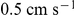 for 300 ms). Subjects then returned their hand to the starting circle, and the cursor representing their hand was only displayed when the hand was within 1.5 cm of the centre of the starting circle. Subjects repeated trials (with a new target selected uniformly subject to its direction from the starting circle being 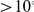 from the preceding target) until they managed to place the centre of the hand cursor within a target circle. They then performed a batch of transformed trials.

Transformed trials were the same as veridical trials except that: 1) a linear transformation was applied between the hand's final location and the displayed cursor position and this transformation was kept fixed within a batch; 2) the position of the visual target (3 cm radius) had to satisfy an added requirement not to overlap the cursor position of the preceding trial; 3) to end a batch subjects had to complete at least three trials and place the centre of the hand cursor within a target circle, and 4) starting on the eighth trial, a batch could spontaneously terminate with a probability of 0.2 after each trial.

For the transformed trials the cursor position (**v**) was a linear transformation (specified by transformation matrix **T**) of the final hand position (**h**) relative to the starting circle: 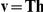. In component form, this can be written:
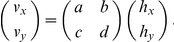



The target color, yellow or blue, indicated whether the trial was veridical or transformed respectively. Subjects were told that on ‘blue’ trials the feedback was not of their actual hand position, but was related to their hand position by a rule. Subjects were told to attempt to learn, and compensate for, this rule in order to hit the targets, and that the rule would be constant across trials until they had hit a target and a set of ‘yellow’ trials had begun. They were told that a new rule was chosen each time a new set of blue trials started, and was unrelated to the rule of the previous set.

#### Second and third sessions

In the second and third sessions, subjects again alternated between making reaching movements under veridical and transformed feedback. However, in the transformed feedback batches, full-feedback trials were included in which the transformed hand cursor was continuously displayed throughout the trial, in order to speed up learning of the transformations and thus of the distribution of transformations. On these trials the batch did not terminate on reaching the target (1 cm radius) and these trials occurred randomly after the third trial with probability 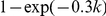, where 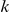 is a trial counter that starts at 1 on the fourth trial and resets to 0 after a full-feedback trial. Thus this probability rises with each consecutive terminal-feedback trial, and drops to zero on the trial after a full-feedback trial.

#### Correlated distribution of transformations

To sample a transformation from the correlated distribution used in sessions 2 and 3, elements 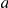 and 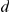 of the transformation matrix were sampled from the uniform distribution 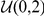. Elements 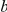 and 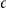 were set equal to each other and were sampled from a zero-mean Gaussian distribution with standard deviation 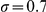. To ensure that the target was reachable, a proposed transformation was then rejected and resampled if it mapped the hand cursor for any hand position within the target rectangle outside the central 80% of either dimension of the 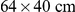 screen, or if it required the hand position to be further than 30 cm from the starting circle to hit any possible target. The resulting distribution of transformations is shown in the top-right cell of Figure 5. This distribution was chosen based on pilot experiments which suggested that subjects have a prior that 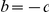 and hence setting 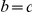 would differ from this prior and engender new learning.

#### Uncorrelated distribution of transformations

In Session 1, the transformation on the first trial was also selected from the correlated distribution. This ensured that the distribution of evidence given to the subject on the first trial was consistent across sessions. However, on the second trial of a batch a new transformation consisted with the first-trial evidence was chosen, and then used for this and all remaining trials of the batch. This new transformation is treated in our analysis as if it had been the transformation throughout the batch, since it would have generated the same evidence on the first trial as the transformation from the correlation distribution. The new transformation was chosen such that across batches there were negligible correlations between any pair of elements in the eventual transformation matrices. To achieve this, at the start of the second trial elements 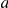 and 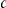 were drawn from Gaussians with 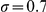 and means 1 and 0 respectively, and 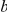 and 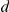 were then uniquely specified so as to be consistent with the hand and cursor positions of the first trial. The rules for rejection of proposed transformations from the correlated distribution were also applied to the choosing of an uncorrelated transform on the second trial of a batch in Session 1; if transformations failed, more were drawn until an eligible transform consistent with the first trial evidence was found. The resulting uncorrelated distribution of the transformation matrices of the second and subsequent trials of the transformed batches of Session 1 (Figure 5, top-left cell) shows minimal correlations between the four elements of the matrix (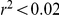 across all pairs), while each element of the matrix has similar standard deviation to in the correlated distribution (Table 2).

**Table 2:** Statistics of the two distributions of transformations.

		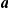	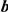	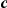	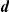
Correlation in uncorrelated distribution	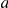	1.00	0.13	0.05	0.13
	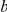		1.00	−0.09	0.03
	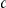			1.00	0.01
	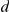				1.00
S.D. in uncorrelated distribution		0.64	0.62	0.72	0.53
S.D. in correlated distribution		0.53	0.54	0.54	0.41
Mean in uncorrelated distribution		1.12	0.01	−0.01	1.07
Mean in correlated distribution		1.17	0.03	0.03	0.99

Top: statistics of the ‘uncorrelated’ and ‘uncorrelated’ distributions, estimated from the 1130 transforms used in Session 1 and the 1091 transforms used in Session 2 respectively.

### Modelling

#### The standard model

Our observer model starts each transformation batch within an experimental session with the same prior probability distribution over transformations. Over the course of each batch, it optimally combines this prior with the evidence shown to the subject, and on each trial uses the updated distribution to select its final hand position.

We vectorize the transformation matrix, i.e. 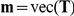, in order to model the probability distribution over transformations as a multivariate Gaussian 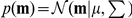. This distribution on the first trial of a transformation batch is the prior, 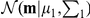. The prior mean is the identity transform: 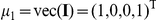. Our inference problem is to the determine the 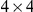 prior covariance matrix 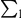. For mathematical simplicity, we actually performed inference on the precision matrix 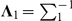.

On any transformed trial 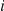 of a batch, the subject has access to the actual (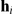) and transformed visual location of the hand (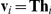). Our observer can use Bayes rule to update its distribution over transformations with this new evidence:
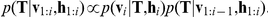



Our aim is to find the prior 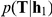, which we can replace with 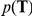 since it is reasonable to assume that the subject does not believe the transformation 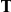 to depend on the first-trial hand position. The likelihood function is:
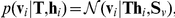



since for tractability we model the internal representation of the hand position 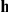 as noiseless, with all noise being on the transformed hand position 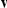 (although in reality this noise consists of two components affecting both 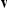 and 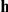). Thus the model observer's probability distribution over the actual 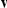, given the 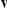 it observes, is 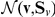, where 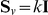. This noise, actually representing both motor and visual noise, was modelled as isotropic Gaussian because a preliminary experiment with unperturbed reaching movements found the combined motor and visual noise in this paradigm to be near to isotropic.

We now express the likelihood function in terms of the vectorized transformation matrix (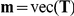):
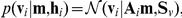



where 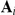 is a function of 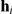:
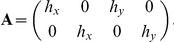



We multiply this Gaussian likelihood with the Gaussian distribution over transformations to give an updated distribution over transformations [58]:
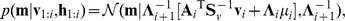



where
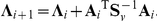



The observer then takes the MAP estimate of the transformation (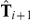) and applies its inverse to the target position on the next trial 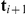, such that the predicted hand position is 
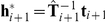
.

It can be shown that scaling the visual noise constant, 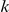, will simply induce the same scaling in the prior covariance 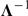 on all trials, with no effect on the predicted hand positions on the second and subsequent trials. Since our analysis focusses on the shape rather than the absolute size of the prior covariance, we simply set 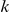 to 1 cm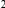.

#### Fitting the model

For a given prior covariance over the elements of the transformation matrix, the model predicts the optimal locations for the reaches on the second trial of each batch (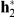). As a measure of goodness-of-fit we computed a robust estimate of error between the predicted and actual hand position (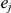 is the Euclidean error on trial 2 of transformation batch 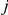) across the 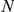 batches of a session for one subject,
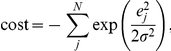



with 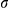 set to 10 cm. Use of this robust error measure reduces sensitivity to outliers. Our choice of 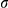 was in order to maximize sensitivity to errors in the 4–10 cm range that was common for predictive errors for our model. We found that using different values for 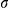 (5 and 20 cm) did not affect our main findings: significantly negative correlation coefficients between 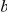 and 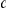 in Session 1 (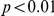 on t-test and Kolmogorov-Smirnov test) that ceased to be significant in Sessions 2 and 3; and significantly negative angles of the 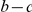 covariance in Session 1 that then clustered around zero and ceased to be significantly negative in Sessions 2 and 3.

We then optimized the covariance matrix for each subject in each session to minimize the cost. We did this by optimizing the 10 free elements of the 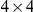 upper triangular matrix 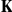, where 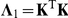. This guarantees that 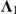 will be symmetric and positive semi-definite (a requirement of a precision or covariance matrix). To further constrain 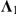, and thus its inverse 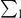, to be positive-definite, the diagonal elements of 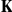 were constrained to be positive. These steps do not prevent near-singular matrices being evaluated; to avoid such numerical problems, 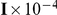 was added to 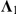 before evaluation of the cost during fitting and at the end of the fitting process.

A trust-region-reflective algorithm implemented by the **fmincon** function of Matlab's Optimization Toolbox was used, with fits started from random precision matrices 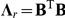, where **B** is a 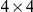 matrix whose elements are independently drawn from a zero mean Gaussian distribution with 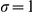. A hundred fits were run for each session and the one with the lowest cost chosen.

#### Validating the model

825 simulated datasets were created by sampling random ‘generating’ priors (created in the same way as the random precision matrices used to initiate model fits) and running the model on an artificial experiment with 150 transformations chosen as for the real experiments. Zero-mean Gaussian noise of covariance 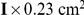 – so chosen to simulate noise from real subjects – was added to the cursor positions.

**Figure 9:**
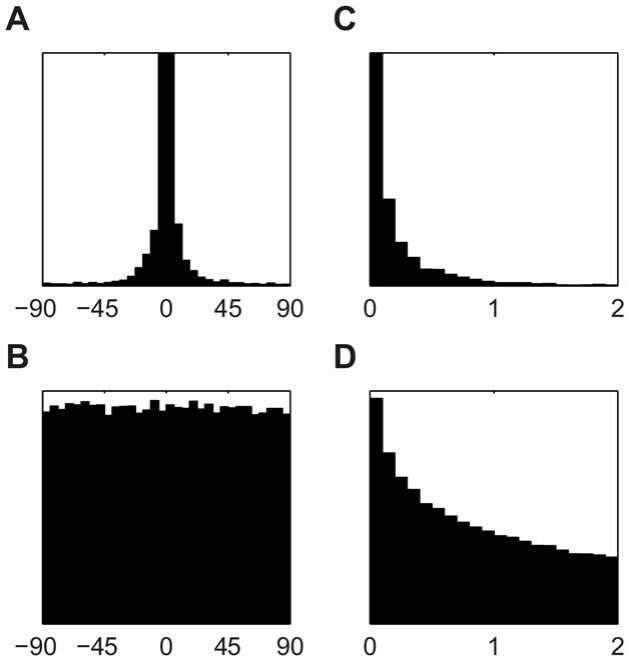
Model validation. (A) The distribution of the difference in covariance orientation angle between pairs of elements in the generating and fitted priors, aggregated across all six pairings of elements. (B) The corresponding distribution when random priors are compared. (C) The distribution of the absolute difference in correlation coefficient between pairs of elements in the generating and fitted priors, aggregated across all six pairings of elements. (D) The corresponding distribution when random priors are compared.

The model was fit to each of these datasets by taking the best of 100 fits. These best fits always gave a lower cost than did the generating prior, due to the finite sample size of the artificial data set. Since our analysis of priors concentrates on the covariance orientation angles and correlation coefficients between pairs of elements, we sought to establish that the differences between these statistics in the generating and fitted priors were small. The median absolute difference in covariance angle between the generating prior and the fitted prior was 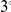 (Figure 9A), compared to 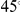 when comparing two randomly-generated priors (Figure 9B). Likewise, the median absolute difference in correlation coefficient between the generating prior and the fitted prior was 0.09 (Figure 9C), compared to 0.72 for random priors (Figure 9D). The fitted correlation was of the wrong sign in 10% of cases, compared to 50% for random priors.

#### Model variations

The standard Bayesian observer model described above correctly assumes the cursor position to be at a linear transformation of the hand position, 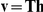. Three other observer models, using the same Bayesian principle but making different assumptions about the transformation, were developed.

The ‘shift’ model assumes the cursor position to be at a shift of the hand position, 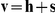. The mean shift in the prior 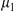 is set at zero. The update equations for the distribution 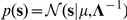 are 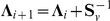 and 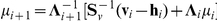. To select its next hand position, the model applies the inverse of the mean shift 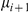 to the target position, such that the predicted hand position is 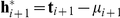.

The ‘rotation & scaling’ model assumes transformations to consist of a rotation and uniform scaling. This was implemented in polar coordinates centred on the start position, as a shift by 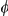 of the angular coordinate and a multiplication by 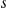 of the radial coordinate. This can be written as,
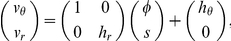



or in vector form, 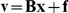. The mean transformation in the prior 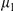 has zero rotation and a scaling gain 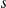 of unity. The update equations for the distribution 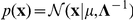 are 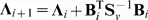 and 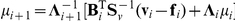. The visual noise covariance 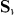 was diagonal, with radial variance 1 cm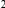 and angular variance 0.1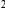, designed to be isotropic at an eccentricity of 10 cm (as in the standard model we fix the magnitude of the variance - see above). The model selects its hand positions using the MAP transformation: 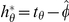 and 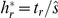.

The ‘affine transformations’ model is the most general of all, assuming the hand position to be subject to a linear transformation and a shift, 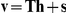. As for the standard model, the transformation equation can be linearized to 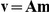, where 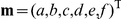 and
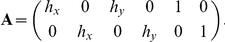



The mean transformation is 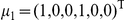, and the update equations are identical to those for the standard model. The MAP transformation 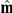 is converted into its linear and shift parts 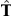 and 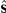, for the purpose of choosing the model hand position on the next trial: 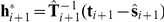. The 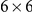 Gaussian distribution over the parameters of the affine transformation did not have covariance between the linear and shift parameters, i.e.
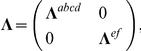



in order to restrict the number of free parameters to 13 (rather than a possible 21).

The same trust-region-reflective algorithm as for the standard model was used to fit the affine model. A slower active-set algorithm, also implemented by the fmincon function of Matlab's Optimization Toolbox, was used to fit the shift and rotation & scaling models; the choice of optimization method was not so important when fitting these models, which have fewer parameters.

Models were compared on the basis of errors between the predicted and actual hand positions. These predictive errors were capped at 20 cm to minimize the effect of outliers, then averaged across all transformations within an experimental session, and then across all subjects and sessions. For trials 3–7 of transformed batches, the Bayesian observer models used priors fit to the second trial of all transformation batches. For comparing prediction errors on the second trial itself, 10-fold cross-validation was used so that complex models did not benefit from over-fitting. The transformations experienced by a subject in one session were assigned into 10 non-overlapping and evenly-spaced groups. For example, if the session included 111 transformations, group 1 consisted of transformations 1, 11, 21, ..., 101, 111; group 2 consisted of transformations 2, 12, 22, ..., 92, 102, etc. Second-trial hand positions were predicted for each group using priors fit as normal to the other nine groups.
